# Supplementary material for: Physical Training Protocols for Improving Dyspnea and Fatigue in Long COVID: A Systematic Review with Meta-Analysis
Source: Healthcare (Basel). 2025 Aug 4;13(15):1897. doi: 10.3390/healthcare13151897 (PMC12346511; doi:10.3390/healthcare13151897)
Supplement: Supplementary file 1 [file healthcare-13-01897-s001.zip › healthcare-3716308-supplementary.pdf]

Physical Training Protocols for Improving Dyspnea and Fatigue in Long COVID: A Systematic Review with Meta-Analysis

Suplementary File. Detailed description of the studies.

| Reference and Country | Title | Author | Study Design | Objective | Sample Size/Age /Sex | Evaluation Instruments | Group | Time/Type of intervention | Statistical Analysis | Main Results | Conclusion | Limitations | Effects on Fatigue and Dyspnea |
|---|---|---|---|---|---|---|---|---|---|---|---|---|---|
| ARAÚJO, B. T. S. et al. Effects of continuous aerobic training associated with resistance training on maximal and submaximal exercise tolerance, fatigue, and quality of life of patients post-COVID-19. Physiotherapy Research | **Effects of continuous aerobic training associated with resistance training on maximal and submaximal exercise tolerance, fatigue, and quality of life of patients post-COVID-19.** | Araújo B.T.S. et al., 2022. | Quasi-experimental. Between March and September 2021. | Evaluated the effects of a cardiopulmonary rehabilitation program consisting of continuous moderate-intensity aerobic and resistance training on lung function, respiratory muscle strength, | N=26 adults >18 years of both sexes. | Digital manometer MVD-300; portable spirometer; cardiopulmonary exercise test (CPET); six-minute walk test; Fatigue Pictogram; Short-form-36; Patient Global Impression of Change. | 1 group pre- and post-intervention analysis | 12 sessions of outpatient intervention. The exercise was performed on a treadmill for 40 minutes: 5 minutes of warm-up, 30 minutes of conditioning, and 5 minutes of cool-down. The resistance training | The Shapiro-Wilk test verified the data distribution. The paired t-test was used to analyze within-group variables before and after the intervention. The McNemar test analyzed categorical variables | This program improved muscle strength, respiratory function, lung function, exercise tolerance, fatigue, and quality of life. Significant improvements were noted in peak VO2, peak VO2 workload, | A cardiopulmonary rehabilitation program consisting of continuous moderate-intensity aerobic and resistance training is effective for post-COVID-19 patients. | The functional deficiencies of many patients during the initial screening, the reduced number of patients, and patients' insecurity about attending or using public transportation to reach the hospital | Fatigue: 36% "a little tired," 32% "moderately tired," 12% "I can do everything I normally do" PRE. POST: 36% "not at all tired" and 28% "I can do everything I normally do." |

| | | | | | | | | | | | | | |
|---|---|---|---|---|---|---|---|---|---|---|---|---|---|
| International, [s. l.], v. 28, n. 1, p. e1972, 2023. Disponível em: https://onlinelibrary.wiley.com/doi/10.1002/pri.1972. Brazil. [17] | | | | maximal and submaximal exercise tolerance, fatigue, and quality of life in post-COVID-19 patients. | | | | consisted of exercises for the upper muscles (triceps, biceps, and shoulder abductors) and lower muscles (quadriceps, hip abductors, and calf muscles). At 60% of one repetition maximum, with load progression every six sessions, twice a week, 3 sets of 8-12 repetitions. | at both time points of the study. Cohen’s d effect size was calculated. | and quality of life, along with a reduction in VE/VCO2 slope and fatigue after the intervention. | | affected the sample size and limited randomization and recruitment of a control group. The hospital stay time could not be stratified. | |

| Reference and Country | Title | Author | Study design | Objective | Sample size/age/sex | Assessment instruments | Group | Time/type of intervention | Statistical analysis | Main results | Conclusions | Limitations | Effects on fatigue and dyspnea |
|---|---|---|---|---|---|---|---|---|---|---|---|---|---|
| BINETTI, J. et al. Clinical and Biomarker Profile Responses to Rehabilitation Treatment in Patients with Long COVID Characterized by Chronic Fatigue. Viruses, [s. l.], v. 15, n. 7, p. 1452, 2023. Disponível em: https://www.mdpi.com/1999-4915/15/7/1452. | **Clinical and Biomarker Profile Responses to Rehabilitation Treatment in Patients with Long COVID Characterized by Chronic Fatigue.** | Binetti, J. et al., 2023. | Study Prospective longitudinal pilot intervention. September 2021 to May 2022. | Analyze the impact of a supervised exercise program on the clinical progression of chronic fatigue syndrome (CFS) in patients with persistent fatigue as the main symptom and identify if certain blood biomarkers could predict the response | N=14 women, average age 44.21 ± 5.50 | Underwent a complete medical history and physical, anthropometric (clinical data), and biochemical examinations. Six-minute walk test; BORG Scale for fatigue and dyspnea; (FIS – Fatigue Impact Scale); (HAD – Hospital Anxiety and Depressio | 2 groups: Group 1 "Responders" Group 2 "Non-responders" to rehabilitation. Those with a 6-minute walk test (6MWT) > 85% performed a series of home rehabilitation exercises (aerobic exercise), while subjects with a 6MWT < 85% were | 3 months; 12 to 20 sessions of supervised physical therapy. Warm-up: stretching and light exercises (5 to 10 minutes); Main: progressive aerobic exercises on a stationary bike (10 to 30 minutes), intensity: based on effort and tolerance from heart rate. One of the weekly sessions included | Kolmogorov-Smirnov test; Student's t-test (parametric variables); Mann-Whitney U test (non-parametric); Chi-square test; Spearman's rho correlation; p-values < 0.05; SPSS 23. | 65% of patients responded to the exercise program, showing improvements in distance walked and oxygen saturation, with stability in the percentage of distance walked and Borg/FIS scales. Participants with obesity and those who were | Patients with chronic fatigue syndrome (CFS) and fatigue demonstrated a partial response but showed a favorable response to a supervised exercise program. | As this is a pilot study (without a control group), the small sample size could reduce statistical power and potentially bias some results; the study duration was limited; results cannot be generalized to the entire population due to the lack of representation of | Dyspnea before: 4.54 ± 2.60; Dyspnea after: 5.44 ± 2.00. Fatigue before: 6.14 ± 1.30; Fatigue after: 6.89 ± 1.90. Note: There was no statistical difference, but there was improvement. |

| | | | | | | | | | | | | | |
|---|---|---|---|---|---|---|---|---|---|---|---|---|---|
| Spain. [18] | | | | to rehabilitation in a cohort from a geographic area with a high prevalence of this condition. | | n Scale); MoCA scale. | included in a supervised exercise program at the hospital rehabilitation service. | exercises for the upper limbs. | | fully vaccinated against SARS-CoV-2 showed a lower degree of fatigue. | | male or older patients. | |
| Reference and Country | Title | Author | Study design | Objective | Sample size/age/sex | Assessment instruments | Group | Time/type of intervention | Statistical analysis | Main results | Conclusions | Limitations | Effects on fatigue and dyspnea |

| | | | | | | | | | | | | | |
|---|---|---|---|---|---|---|---|---|---|---|---|---|---|
| CALVO-PANIAGUA, J. et al. A tele-health primary care rehabilitation program improves self-perceived exertion in COVID-19 survivors experiencing Post-COVID fatigue and dyspnea: A quasi-experimental study. PLOS ONE, [s. l.], v. 17, n. 8, p. e0271802, 2022. Disponível em: https://dx.plos.org/10.1371 | **A tele-health primary care rehabilitation program improves self-perceived exertion in COVID-19 survivors experiencing Post-COVID fatigue and dyspnea: A quasi-experimental study.** | Calvo-Paniagua, J. et al., 2022. | Prospective, multicentric, single-group, and quasi-experimental. From April 2020 to December 2020. At 4 primary care centers in Madrid. | Analyze whether a tele-rehabilitation exercise program is capable of improving self-perceived physical effort in patients with fatigue and dyspnea post-COVID. | Initially, 71 were included, ending with 68 patients aged between 25 and 65 years. Average age 48.5. | 6-minute walk test (6MWT); modified Medical Research Council (mMRC) scale (0–4) and the modified Borg dyspnea scale (0-10). | 1 Group. | 3 months of tele-rehabilitation based on patient education, physical activity, airway clearance, and respiratory exercise interventions were structured into 18 sessions, each lasting 40 minutes, 3 times a week (on alternate days). Therefore, the total duration of the program was planned to be up to 7 weeks. Exercise was conducted via telehealth | SPSS Statistics v.25 $p<0.05$ Shapiro-Wilk test and histograms; Levene's test; normal distribution, or median and interquartile range for variables with non-normal distribution; mixed linear model and Bonferroni correction. | The severity of dyspnea and quality of life improved significantly ($p<0.001$) in self-perception of physical effort during daily activities and were maintained up to 3 months after. | Tele-rehabilitation programs in primary care, based on patient education, physical activity, airway clearance, and respiratory exercises, can be an effective strategy for reducing fatigue and dyspnea post-COVID in COVID-19 survivors. | The design of this study does not include a control group; due to the nature of the tele-rehabilitation program, it was not possible to control some essential aspects of the intervention, such as the intensity of aerobic exercise. The sample size may be considered small. | The severity of dyspnea and reported quality of life significantly improved in self-perception of physical effort. |

| | | | |
|---|---|---|---|
| /journal.pone.0271802. Madrid, Spain. [23] | | through videoconferencing using Zoom; sessions included health education, aerobic training, active mobilizations, and motor control exercises. | |

| Reference and Country | Title | Author | Study design | Objective | Sample size/age/sex | Assessment instruments | Group | Time/type of intervention | Statistical analysis | Main results | Conclusions | Limitations | Effects on fatigue and dyspnea |
|---|---|---|---|---|---|---|---|---|---|---|---|---|---|
| Campos, M. C. et al. Rehabilitation in Survivors of COVID-19 (RE2SCUE): a non-randomized, controlled and open study. medRxiv, p. 2022.10. 10.22280907, 2022. https://doi.org/10.1101/2022.10.10.22280907 Santa Catarina, Brazil. [19] | **Rehabilitation in Survivors of COVID-19 (RE2SCUE): a non-randomized, controlled and open study.** | Campos, M. C. et al., 2022. | Clinical, non-randomized (availability and preference), controlled, and open-label. From April 2021 to April 2022. | Evaluate the effects of an 8-week in-person rehabilitation program for COVID-19 on fatigue and dyspnea, exercise capacity, lung function, cognitive function, symptoms of anxiety and depression, and peripheral muscle strength compared to a remote monitoring group. | 37 participants over 18 years old, both sexes: (n=22; 40.8 ± 10.0 years – in-person) (n=15; 45.4 ± 10.5 years – remote). | Modified Dyspnea Questionnaire of Pulmonary Functional Status; ISWT; pulmonary function tests – spirometer; (HADS); cognition, memory, and attention (RALVT); dynamometer; electrical activity (in microvolts). | 2 groups: 1 in-person rehabilitation group (n=22); 1 remote rehabilitation group (n=15). | 8 weeks, 2 times a week, 80 minutes per session, 75% of initial velocity with BORG 4-6, performed on a treadmill at moderate intensity. Participants completed 5 minutes of warm-up and recovery, and 30 minutes of training at the target intensity. Strength training: starting at 80% of | Comparisons between groups were made using the Shapiro-Wilk test; unpaired t-test; Mann-Whitney U test (for numerical variables); Chi-square test or Fisher's exact test (for categorical variables); pre- and post-intervention within-group results were compared using the paired t-test or | Fatigue was significantly reduced in both the in-person rehabilitation (p=0.0001; d=0.62) and remote monitoring (p=0.012; d=0.41) groups. There was no significant difference between initial fatigue scores (p=0.938) or in the deltas between groups (p=0.292; d=0.17). Dyspnea during | An 8-week in-person rehabilitation program can reduce symptoms of dyspnea, fatigue, and anxiety, increase exercise capacity, and improve memory and attention in patients with persistent COVID-19 symptoms. | Non-randomized; the size of our sample was relatively small, which may have affected the power of intra- and intergroup comparisons. Others reported clinical improvement and expressed interest in not continuing the research; health limitations, lack of social support, lack of motivation | Fatigue was significantly reduced in both the in-person rehabilitation (p=0.0001; d=0.62) and remote monitoring (p=0.012; d=0.41) groups. There was no significant difference in initial fatigue scores (p=0.938) or in the deltas between groups (p=0.292; d=0.17). Dyspnea during activities of daily living |

| | | | | | | |
|---|---|---|---|---|---|---|
| | 10RM with 1–2 minute intervals between 3 sets of 10 repetitions for trunk, upper extremities (UE), and lower extremities (LE), with stretching of trained muscles at the end. For remote rehabilitation, general information on physical activity, breathing exercises, energy conservation techniques, respiratory etiquette, nutritional information, and | Wilcoxon signed-rank test. Delta (Δ) was considered for analyzing between-group outcomes. | activities of daily living (ADLs) was significantly reduced only in the in-person rehabilitation group (p=0.001; d=0.54). There was no difference in baseline dyspnea scores (p=0.901) or in the deltas between groups (p=0.608; d=0.08). Both groups had a significant reduction in the total score of the PFSDQ, with no difference in baseline scores at the start of | | , and financial difficulties are also barriers that impact adherence to pulmonary treatment. | (ADLs) was significantly reduced only in the in-person rehabilitation group (p=0.001; d=0.54). There was no difference in baseline dyspnea scores (p=0.901) or in the deltas between groups (p=0.608; d=0.08). Both groups had a significant reduction in the total PFSDQ score, with no difference in baseline scores at the start of the study (p=0.853) or in the deltas |

| | | | | | | |
|---|---|---|---|---|---|---|
| | | | | water intake was provided. | the study (p=0.853) or in the deltas between groups (p=0.430; d=0.12). | between groups (p=0.430; d=0.12). It can be said that while fatigue improves naturally, dyspnea requires in-person rehabilitation. |

| References and Country | Title | Author | Study design | Objective | Sample size/age/sex | Assessment instruments | Group | Time/type of intervention | Statistical analysis | Main results | Conclusions | Limitations | Effects on fatigue and dyspnea |
|---|---|---|---|---|---|---|---|---|---|---|---|---|---|
| Chikina, S. Y. et al. Effects of physical rehabilitation on exercise tolerance in post-COVID patients: results of an open controlled trial. Pulmonologiya, p. 728-736, 2022. https://doi.org/10.18093/0869-0189-2022-32-5-728-736. Moscou, Russia. [12] | **O impacto da reabilitação física no período pós-Covid Na tolerância ao exercício: resultados de um estudo aberto e controlado.** | Chikina, S. Y. et al., 2022. | Non-randomized, prospective, open-label, and controlled. From 01/03/20 to 31/08/21. | Investigate the effectiveness of physical rehabilitation in the treatment of post-COVID syndrome in patients who have suffered from coronavirus infection with lung damage. | 30 patients, with 26 in the rehabilitation group and 6 in the control group; 14 men and 10 women; GC: 4 men and 2 women Average ages: 51.3 ± 13.1 years and 56.7 ± 10.1 years. | 6-minute walk test (6MWT); severity of dyspnea according to the Borg scale; oxygen saturation (SpO2) measured by pulse oximeter before and after the walk. | 2 groups: intervention and control. | Daily individual sessions at the clinic, including exercises for skeletal muscle using a gym stick, elastic bands, 0.5 to 2 kg dumbbells, and a stepper. Breathing with resistance and exhalation with positive expiratory pressure (respiratory simulators). Before and immediately after | Statistica 12.0 software; Mann-Whitney U test with Yates' correction (for small samples) and 2 × 2 contingency tables; to compare characteristics within groups; Wilcoxon test for paired comparisons; Charlson Comorbidity Index. | There was a significant increase ($p=0.000018$) in resting heart rate, which decreased after exercise ($p=0.017$). SpO2 decreased at rest ($p=0.030$) and after exercise ($p=0.0021$), while dyspnea decreased at the end of the exercise. In the control group, none of the studied indicators changed | Supervised outpatient physical rehabilitation in an ambulatory setting can accelerate physical recovery in post-COVID patients with lung damage. | Firstly, there is the small size of the control sample, which was addressed by using statistical methods for small samples (Mann-Whitney test with Yates' correction). Secondly, there is the non-randomized nature of the study. Randomization was not used for ethical reasons, as it would have required | After exercise ($p = 0.0021$), dyspnea decreased at the end of the exercise; dyspnea on the Borg scale was 0. Dyspnea after walking ($p = 0.017$), oxygen saturation at rest ($p = 0.030$), and after walking ($p = 0.0021$) improved significan |

| | | | | |
|---|---|---|---|---|
| | rehabilitation, the 6-minute walk test (6MWT) was performed according to standard guidelines. In addition to the distance covered in the 6MWT, the intensity of dyspnea was assessed at the beginning and end of the test using the Borg scale, and arterial oxygen saturation (SpO2) was measured with a pulse oximeter. Desaturati | significantly. | excluding patients from the rehabilitation program who wished to exercise and had indications for such training. | tly in the rehabilitation group and did not change significantly in the control group. |

on was defined as a reduction in SpO2 to <90% during exercise or a decrease of ≥4% from the baseline value. Changes in dyspnea, heart rate (HR), and SpO2 before and after the 6MWT were calculated as the difference between initial and final values, represented as Δ of the respective parameter. The control group consisted of patients

| Reference and Country | Title | Author | Study Design | Objective | Sample size/age/sex | Assessment instruments | Group | Time/type of intervention | Statistical analysis | Main results | Conclusions | Limitations | Effects on fatigue and dyspnea |
|---|---|---|---|---|---|---|---|---|---|---|---|---|---|
| | | | | | | | | who attended fewer than 5 sessions or refused physical rehabilitation at the clinic (not related to health). | | | | | |

| | | | | | | | | | | | | | |
|---|---|---|---|---|---|---|---|---|---|---|---|---|---|
| COLAS, C. et al. Management of Long COVID—The CoviMouv' Pilot Study: Importance of Adapted Physical Activity for Prolonged Symptoms Following SARS-CoV2 Infection. Frontiers in Sports and Active Living, [s. l.], v. 4, p. 877188, 2022. Disponível em: https://www.frontiersin.org/articles/10.3389/fspor.2022.877188/full. | **Management of Long COVID—The CoviMouv' Pilot Study: Importance of Adapted Physical Activity for Prolonged Symptoms Following SARS-CoV2 Infection.** | Colas, C. et al 2022. | Pilot, controlled non-randomized. March to July 2021. | Assess fatigue in patients with prolonged symptoms after COVID-19 infection who received a mixed program of adapted physical activity at a distance and therapeutic education. The secondary objective was to evaluate the effectiveness and safety of this training method based on aerobic and anaerobic parameters. | 17 patients (nine men and eight women) were recruited and distributed into tele-R (n = 9) or trad-R (n = 8) groups; Average age: 52.1 ± 12.2 years. | Chalder Fatigue Score (CFS-11); VO2 max; PAM - power at the first ventilatory threshold (VT1); cardiopulmonary exercise test (Vyntus CPX, CareFusion, San Diego, CA, USA); 6-minute walk test (6MWT, in meters); Modified Borg scale. | 2 groups: Personalized rehabilitation (teleR) and traditional physical therapy rehabilitation (control). | 12 sessions of supervised personalized exercises and three therapeutic education workshops for patients with post-COVID-19 fatigue; 3 live sessions of 1 hour and 45 minutes of aerobic exercise and 15 minutes of resistance exercise, conducted at home via videoconference over 3 weeks. Control group: traditional physical rehabilitation with a | Jamovi statistics software. Data were checked for normality and homogeneity of variances using the Shapiro-Wilk and Levene tests, respectively; with Shapiro-Wilk tests, either a two-factor repeated measures ANOVA or Friedman test was conducted. Where significant interaction differences occurred, post hoc Tukey analyses were performed | Fatigue was reduced after one month of intervention in both groups (p = 0.010). Aerobic parameters improved significantly: VO2 max (p = 0.005), Distance covered (p = 0.019), Hyperventilation values (p = 0.035). The anaerobic parameter did not improve (p = 0.400). | Improvement in the CFS-11 in both tele-R and trad-R groups. Managing fatigue through exercise can be an effective solution for these patients with persistent symptoms. Tele-rehabilitation is a good alternative when an in-person program is not possible. | By including patients referred by hospital COVID-19 services and whose COVID-19 infection was confirmed by RT-PCR, a larger study would be necessary to assess the relevance of physical training in the tele-rehabilitation of these patients; long-term follow-up to determine if the benefits of tele-rehabilitation persist | Fatigue was reduced after one month of intervention in both groups (p = 0.010), showing a significant time effect with no difference between the groups. |

| | | | | | | | | | | | | | |
|---|---|---|---|---|---|---|---|---|---|---|---|---|---|
| France.[18] | | | | | | | | community physiotherapist (3 physiotherapy sessions per week for 4 weeks). | with $p < 0.05$. | | | over time. Additionally, the existence of recommendations from major organizations such as the World Health Organization, the National Institute for Health and Care Excellence, and the French High Authority for Health underscores the importance of recognizing and managing symptoms to prevent anxiety and medical errors. | |

| Reference and Country | Title | Author | Study desihn | Objective | Sample size/age/sex | Assessment instruments | Group | Time/type of intervention | Statistical analysis | Main results | Conclusions | Limitations | Effects on fatigue and dyspnea |
|---|---|---|---|---|---|---|---|---|---|---|---|---|---|
| COMPAGNO, S. et al. Physical and psychological reconditioning in long COVID syndrome: Results of an out-of-hospital exercise and psychological - based rehabilitation program. IJC Heart & Vasculature, [s. l.], v. 41, p. 101080, 2022. Disponível em: https://link | **Physical and psychological reconditioning in long COVID syndrome :Results of an out-of-hospital exercise and psychological – based rehabilitation program.** | Compagno, S. et al., 2022. | Cohort study. April 2021 to November 2021. | Assess the efficacy, safety, and feasibility of an extra-hospital multidisciplinary rehabilitation (MDR) program, based on physical and psychological reconditioning, in reducing symptoms and improving physical fitness and psychological parameters in patients with Post-COVID Syndrome (LCS). | 30 participants (18 men, average age of 58 years). | Medical screening including resting electrocardiogram and transthoracic echocardiogram, body composition assessment (Tanita Corporation), muscle strength assessment, cardiopulmonary exercise test (CPET on a cycle ergometer), psychological and quality of life evaluation | 1 group: Pre- and post-intervention analysis. | Individual relaxation techniques, including: muscle relaxation, body scan (meditation exercise - breathing control and imaginative relaxation). Additional program: According to ACSM guidelines: 3 times a week, 90 minutes (10 minutes warm-up; 45 minutes strength training; 5 minutes relaxation; | Shapiro-Wilk (we verified that all outcome parameters had a normal distribution); quantitative variables were summarized as mean (m) and standard deviation (SD), while categorical variables were summarized as absolute values (n) and percentages (%). Paired t-tests; Fisher's exact test; | After the MDR program, residual COVID-19 symptoms decreased significantly. Significant improvements were observed in upper and lower limb muscle strength, cardiopulmonary parameters, physical and mental health perception, depression, and anxiety. | This study confirms the severe physical and psychological impairment in patients with Long COVID Syndrome (LCS) and suggests that a multidisciplinary rehabilitation (MDR) program is effective, safe, and feasible for these patients, potentially promoting their physical and psychological recovery. | Sample size; lack of a control group; we did not differentiate the impact of specific treatment measures such as physical training and psychological interventions but focused on the overall effects of the multidisciplinary rehabilitation strategy; post-COVID syndrome was not measured | Residual symptoms, including dyspnea and fatigue, decreased significantly, with 50% of participants no longer experiencing any symptoms. |

| | | | | | | | | | | | | | |
|---|---|---|---|---|---|---|---|---|---|---|---|---|---|
| inghub.elsevier.com/retrieve/pii/S2352906722001294. Venice, Italy. [20] | | | | | | (QoL), before and after the MDR program, caliper measurements and manual grip at the Cardiovascular Rehabilitation and Sports Medicine Service of the Noale Hospital, Venice, Italy. Leg press test and Zung scales (anxiety and depression). | | followed by 35 minutes of continuous moderate-intensity training (60–80% of peak VO2 from CPET). We did not differentiate the impact of specific treatment measures, such as physical training and psychological interventions, but rather focused on the overall effects of the multidisciplinary rehabilitation strategy. | $p < 0.05$; analyzed using STATA software. | | | with a validated questionnaire, and long-term data is lacking. | |

| Reference and Country | Title | Author | Study design | Objective | Sample size/age/sex | Assessment instruments | Group | Time/type of intervention | Statistical analysis | Main results | Conclusions | Limitations | Effects on fatigue and dyspnea |
|---|---|---|---|---|---|---|---|---|---|---|---|---|---|
| VIEIRA DA COSTA, K. et al. Efficacy of a rehabilitation protocol on pulmonary and respiratory muscle function and ultrasound evaluation of diaphragm and quadriceps femoris in patients with post-COVID-19 syndrome: a series of cases. Monaldi Archives for Chest Disease, | **Efficacy of a rehabilitation protocol on pulmonary and respiratory muscle function and ultrasound evaluation of diaphragm and quadriceps femorisin patients with post-COVID-19 syndrome: a series of cases.** | VIEIRA DA Costa, K. V. et al., 2023. | Case reports. | Efficacy of a pulmonary rehabilitation protocol on respiratory and pulmonary muscle function, as well as on the thickness of the femoral quadriceps and diaphragm in patients with post-COVID-19 syndrome. | 9 cases; 5 women and 4 men, average age of 52.77 years. | Pulmonary function tests, respiratory and diaphragmatic muscle function tests, fatigue resistance index (FRI), and ultrasound of the diaphragm and quadriceps femoris. | Pre- and post-intervention assessment. | Frequency: Twice a week for 6 weeks. Pulmonary Expansion Therapy: Method: Positive End-Expiratory Pressure (PEEP). Protocol: 3 sets of 2 minutes with 1-minute rest intervals. Initial Pressure: Set according to individual needs. Inspiratory Muscle Training: Method: Using a threshold | - (Does not mention). | Fatigue Resistance Index (FRI): Initial Value: 0.87 (reduced for the general population). Post-Intervention Value: 1.03 (considered normal). Pulmonary Function Improvements: Peak Expiratory Flow (PFE): Increased by 26.03%. Forced Vital Capacity (FVC): | PR based on rehabilitation principles reduced quadriceps echo intensity and improved pulmonary function, respiratory muscle strength and endurance, and the thickness of the diaphragm and femoral quadriceps in patients with post-COVID-19 syndrome. | - (Does not mention). | Pulmonary rehabilitation was conducted to reverse respiratory sequelae such as exercise-induced dyspnea, dry cough, chest pain, and fatigue. Our patients showed promising results in pulmonary function, respiratory muscle strength and endurance, and diaphragmatic and peripheral ultrasound after six weeks. |

| | | |
|---|---|---|
| [s. l.], 2022. Disponível em: https://www.monaldiarchives.org/index.php/macd/article/view/2206. Brazil. [11] | inspiratory device. Protocol: 3 sets of 10 repetitions at 40% of Maximum Inspiratory Pressure (MIP). Upper Limb Strength Exercises: Method: Shoulder flexion with weights. Protocol: 3 sets of 1 minute with 1-minute rest between sets. Load: 50% of the test weight, increased by 0.05 kg according to Perceived Exertion Scale (PSE). | Increased by 7.14%. Forced Expiratory Volume in 1 second (FEV1): Increased by 9.55%. FEV1/FVC Ratio: Increased by 6.19%. These improvements indicate a significant enhancement in both pulmonary and respiratory muscle function following the rehabilitation program. |

| | | | | | | | | | | | | | |
|---|---|---|---|---|---|---|---|---|---|---|---|---|---|
| | | | | | | | | Aerobic Exercise: Method: Treadmill training. Protocol: Weeks 1-3: Steady-state at 60-70% of the heart rate obtained from the exercise test. Weeks 4-6: Increased to 70-80% of the heart rate obtained from the exercise test. | | | | | |
| **Reference and Country** | **Title** | **Author** | **Study design** | **Objective** | **Sample size/age/sex** | **Assessment instruments** | **Group** | **Time/type of intervention** | **Statistical analysis** | **Main results** | **Conclusions** | **Limitations** | **Effects on fatigue and dyspnea** |
| DUMITRESCU, A. et al. Post-Severe-COVID-19 Cardiopulmonary | **Post-Severe-COVID-19 Cardiopulmonary Rehabilitation: A** | Dumitrescu, A. et al., 2023. | Prospective cohort analysis, convenience sampling. September 2021 to | Evaluate the impact of COVID-19 on cardiopulmonary health and | 84 patients, with an average age of 56.3 years in the high-intensity | (Chest CT scans; laboratory tests: elevated D-dimer, lymphopenia, | 2 groups (n=42 for each group). | 3 months.<br><br>The low-intensity protocol involved initial training on | Software SPSS: To compare proportions, the Chi-square and Fisher's exact tests | The level of physical activity showed no difference (p = 0.512) | Low and high-intensity cardiopulmonary rehabilitation programs | Single-center study conducted at a specific hospital in Romania; | Did not directly address fatigue. However, parameters such as peak |

| | | | | | | | | | | | | | |
|---|---|---|---|---|---|---|---|---|---|---|---|---|---|
| Rehabilitation: A Comprehensive Study on Patient Features and Recovery Dynamics in Correlation with Workout Intensity. Journal of Clinical Medicine, [s. l.], v. 12, n. 13, p. 4390, 2023. Disponível em: https://www.mdpi.com/2077-0383/12/13/4390. Timisoara, Romania. [12] | **Comprehensive Study on Patient Features and Recovery Dynamics in Correlation with Workout Intensity.** | | September 2022. | assess the effectiveness of various rehabilitation interventions. This study hypothesizes that post-COVID-19 patients exhibit distinct characteristics and recovery dynamics that significantly influence their response to specific rehabilitation interventions. | group and 53.1 years in the low-intensity group. | elevated C-reactive protein (CRP), liver enzymes (AST, ALT), IL-6, and ferritin; respiratory impairment, such as partial pressure of oxygen (PaO2), fraction of inspired oxygen (FiO2) less than 300; acute respiratory distress syndrome (ARDS). | | a cycle ergometer, starting with a load of 20–30 Watts, approximately 40–50% of the patient's estimated maximum effort. The sessions were scheduled three times a week. As the patient's endurance improved, gradual walking exercises were incorporated, initially in indoor environments and then progressing to outdoor settings. Respiratory | were used. For comparing differences between groups with non-parametric data, the Mann-Whitney test was utilized. The Student's t-test was applied for independent and unpaired samples. | between the two groups. This study provides evidence that personalized and targeted rehabilitation strategies can improve cardiopulmonary health in the long term for patients recovering from severe COVID-19. It demonstrates that both low and high-intensity training are effective in enhancing cardiac and pulmonary function | play a crucial role in improving cardiopulmonary parameters. They highlight the importance of implementing comprehensive cardiopulmonary rehabilitation protocols in the treatment of patients with severe post-COVID-19 and underscore the value of intensity-stratified rehabilitation based on individual patient dynamics | the limited sample size (although sufficiently powerful for the analyses performed) may not provide precise estimates of effect size or allow for stratified analyses based on various comorbidities or demographic characteristics; limited sample size; follow-up only for 3 months; absence of a control group. | oxygen consumption, respiratory exchange rate, and respiratory reserve showed significant improvements (dyspnea). |

| | | |
|---|---|---|
| exercises included guided coughing to clear the airways and drainage postures to aid in mucus removal. Strength training involved low-weight resistance exercises using 1–2 kg dumbbells or low-elasticity resistance bands, performed twice a week. Flexibility exercises were done 2 to 3 times a week. Educational sessions were integral to the | when conducted properly and over a period of up to 3 months. | and recovery characteristics. |

protocol, focusing on teaching patients to assess their level of effort. Each exercise session, including warm-up, main exercise, and cool-down, lasted approximately 45–60 minutes.

The high-intensity protocol consisted of more rigorous training. Aerobic exercises on the cycle ergometer were increased to a load of 40–50 Watts, approxima

tely 60–70% of the patient's estimated maximum effort, with a frequency increased to five times a week. For patients showing favorable responses, interval training with alternating periods of increased and reduced effort was introduced. Respiratory exercises were complemented with inspiratory muscle training using a threshold device to improve

respiratory muscle strength and endurance. The resistance in strength training was gradually increased, with training frequency set at 3–4 times a week. For patients demonstrating good progress, high-intensity interval training (HIIT) was cautiously introduced, with monitoring of patient tolerance and response. Educational sessions were

| | | | | | | | | | | | | | |
|---|---|---|---|---|---|---|---|---|---|---|---|---|---|
| | | | | | | | | extended to additional topics, including stress management, coping strategies, and nutritional counseling. | | | | | |
| Reference and Country | Title | Author | Study design | Objective | Sample size/age/sex | Assessment instruments | Group | Time/type of intervention | Statistical analysis | Main results | Conclusions | Limitations | Effects on fatigue and dyspnea |
| Elhamrawy, M. Y. et al., 2023. Effect of Tai Chi versus aerobic training on improving hand grip strength, fatigue, and functional performance in older adults post-COVID-19: a | **Effect of Tai Chi versus Aerobic Training on Improving Hand Grip Strength, Fatigue, and Functional Performance in Older Adults Post-COVID-** | Elhamrawy, M. Y. et al., 2023. | Clinical Trial Randomized. September 2022 to January 2023. | To understand the impact of TC (Traditional Care) versus AT (Active Therapy) on Handgrip Strength, fatigue, and functional performance in elderly patients | 54 participants. | Handgrip Strength (FPM)<br>Fatigue Severity Scale (FSS)<br>30-Second Chair Stand Test (CST-30)<br>30-Second Arm Curl Test (ACT)<br>8-Foot Up-and-Go Test (TUG) | 3 Groups:<br>TC (Tai Chi);<br>AT (Aerobic Training);<br>CON (Control). | Duration: 3 months<br>TC (Tai Chi): 4 sessions of 60 minutes per week for 12 consecutive weeks<br>AT (Aerobic Training): 4 sessions of 60 minutes per week<br>CON (Control): | Software Used: SPSS<br>Statistical Test: ANOVA for comparisons between groups. | Both Tai Chi (TC) and Aerobic Training (AT) showed significant improvements in all evaluated parameters. Specifically, Tai Chi demonstrated a significant improvement in: | Tai Chi is an integrated rehabilitation program that positively affected grip strength, fatigue levels, and functional performance compared to aerobic training in older | - | After the intervention, both the Tai Chi (TC) and Aerobic Training (AT) groups experienced significant improvements in grip strength (FPM), fatigue levels, 30-second chair stand |

| | | | | | | | | |
|---|---|---|---|---|---|---|---|---|
| randomized controlled trial. Journal of Population Therapeutics and Clinical Pharmacology, [s. l.], v. 30, n. 7, 2023. Disponível em: https://www.jptcp.com/index.php/jptcp/article/view/1591/1732. Beni-Suef, Egypt. [30] | **19: a randomized controlled trial.** | | post-COVID-19. | | Advised to maintain their usual daily routine. | Grip Strength: p=0.0435, with a mean difference of -2.5 Arm Curl Test (ACT): p=0.0235, with a mean difference of 1 These results indicate that Tai Chi provided additional benefits compared to Aerobic Training in these specific measures. | adults post-COVID-19. | test (CST), 30-second arm curl test (ACT), 8-foot Up-and-Go test (TUG), and 2-minute walk test compared to baseline (all p < 0.001). For fatigue measured by the Fatigue Severity Scale: TC: 44.9 ± 3.8 (p = 0.0001) and AT: 43.7 ± 2.1 (p = 0.0001). |

| Reference and Country | Title | Author | Study design | Objective | Sample size/age/sex | Assessment instruments | Group | Time/type of intervention | Statistical analysis | Main results | Conclusions | Limitations | Effects on fatigue and dyspnea |
|---|---|---|---|---|---|---|---|---|---|---|---|---|---|
| ESPINOZA-BRAVO, C. et al. Effectiveness of Functional or Aerobic Exercise Combined With Breathing Techniques in Telerehabilitation for Patients With Long COVID: A Randomized Controlled Trial. Physical Therapy, [s. l.], v. 103, n. 11, p. pzad118, 2023. Disponível em: https://academic.oup.com/ptj/ar | **Effectiveness of Functional or Aerobic Exercise Combined With Breathing Techniques in Telerehabilitation for Patients With Long COVID: A Randomized Controlled Trial.** | Espinoza-Bravo, C. et al., 2023. | Randomized, double-blind, controlled clinical trial conducted from November 2021 to May 2022. | Compare the short-term clinical effects of two tele-rehabilitation programs—functional exercises versus aerobic exercises (AEs), both combined with respiratory techniques—on improving symptoms of post-COVID-19 syndrome (long COVID). | 43 participants, aged between 20 and 60 years; Functional Exercises (FE) group (n=21); Aerobic Exercises (AE) group (n=22); mean age = 42.4 [SD = 6.5] years. | Assessments conducted at baseline and post-intervention:<br>Fatigue: Fatigue Severity Scale (FSS) Dyspnea: London Chest Activity of Daily Living Scale (LCADL) Functional Performance: 30-Second Sit-to-Stand Test Perceived Stress: Perceived Stress Scale (PSS) Anxiety and | Study Groups: Functional Exercise (FE) Aerobic Exercise (EA) Both groups included breathing techniques. | Intervention Details:<br>Duration: 8 weeks Frequency: 3 sessions per week Platform: Mobile app Fisiotrack Evaluations: Timing: Conducted at baseline and post-treatment | Normality Check: Shapiro-Wilk test Baseline Comparison: Categorical Variables: Chi-square test ($\chi^2$) Continuous Variables (Normal Distribution): Student's t-test Non-parametric Variables: Mann-Whitney U test Mixed-Design ANOVA: For time-based changes in fatigue and dyspnea | Intragrupo Comparisons: Functional Exercise (FE) Group:<br>Fatigue: Improvement of -6.7 points (95% CI = -11.9 to -1.3) Functional Capacity: Improvement of 2.6 repetitions (95% CI = 0.3 to 4.9) Perceived Stress: Improvement of -4.9 points (95% CI = -9.1 to -0.8) Aerobic Exercise (AE) Group: | Both tele-rehabilitation exercise modalities are effective in improving stress symptoms and quality of life in patients with long-term COVID-19. To improve fatigue and functional performance, Functional Exercise (FE) shows more promising results. | Absence of a control group (which was not considered in this study, as almost the entire target population had no access to rehabilitation and had been waiting for months for an alternative treatment to manage their symptoms); short-term intervention; use of self-reported assessments and the manner in | FE showed improvement in fatigue (-6.7 points; 95% CI = -11.9 to -1.3). Significant differences were observed within the FE group for fatigue, functional performance, and perceived stress. Additionally, fatigue and quality of life exhibited clinically significant improvements after treatment in the FE group (-4 and -0.051, respectively). In the |

| | | | | | | | | | | | | | |
|---|---|---|---|---|---|---|---|---|---|---|---|---|---|
| ticle/doi/10.1093/ptj/pzad118/7258918. Valencia, Spain. [30] | | | | | | Depression: Hospital Anxiety and Depression Scale (HADS) Quality of Life: European Quality of Life Scale (EQ-5D) Perceived Change Post-Treatment: Patient Global Impression of Change Scale (PGI-C) System Usability: System Usability Scale (SUS) for the Fisiotrack mobile app Treatment Adherence was also examined | | | Software: SPSS version 22.0 Significance Level: $p < 0.05$. | Perceived Stress: Improvement of -6.2 points (95% CI = -10.3 to -2.1). | | which treatment adherence was recorded may be limitations. | AE group, significant differences were found within the group for perceived stress, and clinically significant improvements were noted for fatigue, quality of life, and depression symptoms (-4, -0.051, and -1.6, respectively) after treatment. |

post-
treatment.

| Reference and Country | Title | Author | Study design | Objective | Sample size/age/sex | Assessment instruments | Group | Time/type of intervention | Statistical analysis | Main results | Conclusions | Limitations | Effects on fatigue and dyspnea |
|---|---|---|---|---|---|---|---|---|---|---|---|---|---|
| HASENOEHRL, T. et al. Post-COVID: effects of physical exercise on functional status and work ability in health care personnel. Disability and Rehabilitation, [s. l.], v. 45, n. 18, p. 2872–2878, 2023. Disponível em: https://www.tandfonline.com/doi/full/10.1080/09638288.2022.2111467. | **Post-COVID: effects of physical exercise on functional status and work ability in health care personnel.** | Hasenoehrl, T. et al., 2022. | Randomized, single-blind, controlled trial conducted from May to July 2021. | Evaluate the effects of physical exercise on post-COVID-19 symptoms, physical/ mental capacities, and work capacity within the scope of a workplace health promotion project for healthcare professionals. | 32 participants (health professionals enrolled); 42.9 years; and 47.4 years among those with severe symptoms; 60.0% in the moderate symptom group (MSG) and 89% in the severe symptom group (SSG). | The primary outcome parameter for physical fitness was peak VO2, in addition to TC6M (physical function), 30secSTS, (GAD-7 anxiety), (PHQ-9 depression), (PSS-10 stress), fatigue (BFI), (BRS resilience), (MoCA cognitive), (WAI workability). Assessment at baseline, after 4 weeks, | 2 groups: Severe and moderate based on Post-COVID Functional Scale (PCFS) scores: (1) severe symptoms (SSG, $n = 11$) and (2) mild symptoms (MSG, $n = 21$). | Participants underwent an 8-week exercise intervention program consisting of two supervised sessions of resistance exercises per week plus individual recommendations for aerobic exercises. | Hypothesis tests (for differences in medians) employed the Mann-Whitney U test; association was estimated both via Pearson coefficient (performance parameters, PCFS, WAI) and Spearman's Rho (with respect to psychological scores), and distribution differences were tested via Chi- | VO2 peak improved significantly in the SSG by 2.4 ml/kg/min (95% CI [1.48; 3.01], adjusted $p < 0.001$) and not significantly in the MSG by 1.27 ml/kg/min (adjusted $p = 0.096$). Both groups significantly improved their 30-second STS ($p = 0.0236$) and 6MWT ($p = 0.0252$) results at both | Cases with severe fatigue showed higher levels of benefit compared to those with mild symptoms. Physical exercise proved to be an effective intervention method in the rehabilitation of COVID-19 survivors suffering from post-COVID syndrome, positively affecting both physical and mental | Lack of an inactive control group; we could not analyze the home-based aerobic exercise program in detail; however, the analysis of step counts indicated similar levels of physical activity in both groups. | Cases with severe fatigue showed higher levels of benefit compared to those with mild symptoms. |

| | | | | | | | | |
|---|---|---|---|---|---|---|---|---|
| Áustria. [15] | | | and at the end of the exercise intervention (8 weeks). | | | Square tests modified for multiple variables. Metric variables (6MWT, 30-second STS test, and peak VO2 relative) were analyzed at different follow-up periods and across different groups (MSG vs. SSG) using mixed-effects repeated measures models with Geisser-Greenhouse correction for sphericity and Sidak-corrected | follow-ups (4 weeks and 8 weeks after inclusion). The SSG improved more than the MSG in VO2 peak and 6MWT, both after 4 and 8 weeks, respectively, although not statistically significant; results were the opposite for the 30-second STS. The results of the 30-second STS correlated significantly with mental health and workability | health in healthcare professionals experiencing post-COVID syndrome. Increases in physical performance are directly related to improvements in workability. |

| | | | | | | | | | | | | | |
|---|---|---|---|---|---|---|---|---|---|---|---|---|---|
| | | | | | | | | | post hoc tests. GraphPad Prism and SPSS v27, α = 0.05. | y outcomes. | | | |
| References and Country | Title | Author | Study design | Objective | Sample size/age/sex | Assessment instruments | Group | Time/type of intervention | Statistical analysis | Main results | Conclusions | Limitations | Effects on fatigue and dyspnea |
| JIMENO-ALMAZÁN, A. et al. Rehabilitation for POST-COVID - 19 condition through a supervised exercise intervention: A randomized controlled trial. Scandinavian Journal of Medicine & Science in Sports, [s. l.], v. 32, n. 12, p. | **Rehabilitation for post-COVID-19 condition through a supervised exercise intervention: A randomized controlled trial** | Jimeno-Almazán, A. et al., 2022b. | Randomized clinical trial that included 39 participants with post-COVID-19 condition who had a chronic symptomatic phase lasting >12 weeks. | Compare the outcomes of patients with post-COVID-19 condition undergoing supervised therapeutic exercise intervention versus those following the WHO (World Health Organization) self-management rehabilitation leaflet. | 39 participants, average age 45.2 years, with 74.4% being female (n = 29). | Short Form Survey of 12 items (SF-12); Cardiopulmonary exercise test (Ergoline, Ergoselect 200) using heart rate monitors (Polar V800, Kempele, Finland) and reporting perceived exertion (RPE 6-20) according to the Ekblom- | 2 groups: Group 1: Multicomponent intervention. Group 2: Followed WHO guidelines (control group). | Patients were randomly assigned to either a personalized multicomponent exercise program based on concurrent training for 8 weeks (two supervised sessions per week consisting of resistance training combined with aerobic | The independence of groups at the start of the study was assessed using the t-test. Analysis of Covariance (ANCOVA) was conducted to determine if post-test values in health markers and symptoms differed between groups. | After the follow-up, changes in physical outcomes were observed in both groups; however, the magnitude of change from pre- to post-intervention favored the exercise group in cardiovascular and strength markers: VO2 max increased by 5.7%, sit-to- | Compared to the current WHO recommendations, a supervised, personalized concurrent training program with low to moderate intensity is more effective, safe, and well-tolerated for post-COVID-19 conditions. | As this study represents a limited-sized outpatient population, the results may not be directly applicable to other cohorts with post-COVID-19 conditions or sequelae, particularly those with moderate to severe acute SARS- | The exercise intervention resulted in significantly improved quality of life, reduced fatigue, lower depression, and better functional status, as well as superior cardiovascular fitness and muscle strength compared to controls ($p < 0.05$). The exercise |

| | | | | | | | | | | | | | |
|---|---|---|---|---|---|---|---|---|---|---|---|---|---|
| 1791–1801, 2022. Disponível em: https://onlinelibrary.wiley.com/doi/10.1111/sms.14240. Spain. [25] | | | | | | Bak protocol; Digital dynamometer (Takei 5401-C, Shinagawa-Ku, Tokyo); Sit-to-stand test; Isometric knee extension test (Chronojump, BoscoSystem, Barcelona) recorded in Newtons (N); Submaximal progressive load test (Smith machine); Linear velocity transducer (T-Force, Ergotech Consulting, Murcia, Spain); WHO Global | | training [moderate-intensity variable training], plus a third day of monitored continuous light-intensity training), or to a control group following WHO guidelines for post-COVID-19 rehabilitation. The strength training included: 50% 1RM, 3 sets, 8 repetitions, with 4 exercises [squats, bench press, deadlift, and lat pulldown]. | Chi-square tests and factorial ANOVA 2 (group: RECOVE vs. CONTROL) × 2 were also employed. Bonferroni adjustments were applied, with significance set at p<0.05. | stand performance improved by 22.7%, and load-velocity profiles in bench press increased by 6.3% and in half squat by 16.9% (p < 0.05). The exercise group reported improvements in all physical variables examined. The STS test and HSQ improved in both groups. | | CoV-2 infection requiring hospitalization. Additionally, the Ekblom-Bak test protocol may fail to accurately estimate VO2 max in individuals with extreme reductions in physical fitness or significant limitations in heart rate. | group showed significant changes in fatigue (SF-12, bimodal and Likert CFQ-11, FSS, and PCSF), with more pronounced effects after the intervention, especially in reported dyspnea (controls vs. exercise: 83.3% vs. 5.4%, p = 0.003; V = 0.48) and fatigue (61.1% vs. 34.6%, p = 0.072; V = 0.30). |

Physical Activity Questionnaire (GPAQ); Resting ECG and Echocardiogram performed following standard procedures; Forced spirometry test (MetaLyzer 3B-R3, Cortex Biophysik GmbH, Leipzig, Germany); Forced Vital Capacity (FVC); Maximum Voluntary Ventilation (MVV). Modified Dyspnea Scale (mMRC).

| Reference and Country | Title | Author | Study design | Objective | Sample size/age/sex | Assessment instruments | Group | Time/type of intervention | Statistical analysis | Main results | Conclusions | Limitations | Effects on fatigue and dyspnea |
|---|---|---|---|---|---|---|---|---|---|---|---|---|---|
| SMITH, J. L. et al. Improved clinical outcomes in response to a 12-week blended digital and community-based long-COVID-19 rehabilitation programme. Frontiers in Medicine, [s. l.], v. 10, p. 1149922, | **Improved clinical outcomes in response to a 12-week blended digital and community-based long-COVID-19 rehabilitation programme.** | Smith, J. L. et al., 2023. | Mixed design and scalable community-based study February 2021 to March 2022. | Evaluate the clinical efficacy of a new 12-week combined community rehabilitation program for individuals with Long COVID (LC). | 601 adult participants with Long COVID who underwent the Nuffield Health COVID-19 Rehabilitation Program. | Shortness of breath was measured using the Dyspnea-12 (D-12) tool; Functional capacity was assessed using the Duke Activity Status Index (DASI); Physical fitness was evaluated with the 30-second Sit-to-Stand | 1 group (intervention). A complete case analysis was performed for the 601 participants who completed the Nuffield Health COVID-19 Rehabilitation Program. | 12-week program 3 exercise sessions per week, including aerobic and strength exercises, stability, and mobility activities. The first 6 weeks were conducted remotely, and the last 6 weeks included in-person rehabilitation | Paired t-tests were used to determine significant differences between time points. Two-sided 95% confidence intervals were used for all analyses. A 5% alpha level was applied. All analyses were performed using R | Significant improvements were observed in the Dyspnea-12 (D-12) scores, Duke Activity Status Index (DASI), World Health Organization-5 (WHO-5), and EQ-5D-5L (all $p < 0.001$).<br><br>At the end of the program, a | This assessment demonstrated significant and clinically meaningful improvements in dyspnea, functional capacity, mental well-being, and quality of life scores in response to a 12-week rehabilitation | No control group was included. The majority of participants were female (77.4%) and of white British ethnicity (88.6%). Specific population measures like the Post-COVID Functional Status Scale (PCFS) | Significant Improvement: There was a significant improvement in the Dyspnea-12 (D-12) scores ($p < 0.001$). |

| | | | | | | | | | | | | |
|---|---|---|---|---|---|---|---|---|---|---|---|---|
| 2023. Disponível em: https://www.frontiersin.org/articles/10.3389/fmed.2023.114992/full. Europe; África e Àsia. [23] | | | | | | Test; Mental well-being was measured using the World Health Organization Five Well-being Index (WHO-5); Health status was assessed using the EuroQoL Five Dimension Five Level (EQ-5D-5L) and the Visual Analogue Scale (VAS). | | sessions in a community setting. Sessions: Group rehabilitation exercise session lasting 45 minutes. On-demand exercise session lasting 45 minutes, using pre-recorded guided sessions available on an online platform (Vimeo, New York, USA). Self-directed exercise session, where participants selected activities from a provided rehabilitati | version 4.1.2. | reduction in medical consultations, sick leave, and outpatient hospitalizations was also noted compared to the previous 3-month period. These findings suggest that the Nuffield Health COVID-19 Rehabilitation Program may address the urgent need for an effective and scalable LC rehabilitation model. | program for participants with Long COVID. These findings suggest that the Nuffield Health COVID-19 Rehabilitation Program may fulfill the urgent need for an effective and scalable LC rehabilitation model. | were not used. The rehabilitation was monitored over a relatively short period of 12 weeks. |

| |
|---|
| on booklet to create a session suited to their personal limits. Sessions included: 12 weeks total (6 weeks remote and 6 weeks in-person) 3 sessions per week (36 in total), including one group session, one pre-recorded session, and one self-directed session. Activities included a combination of cardiovascular, strength, and mobility exercises. |

| | | | | | | | | | | | | | |
|---|---|---|---|---|---|---|---|---|---|---|---|---|---|
| | | | | | | | | Intensity and volume of exercise, as well as movement complexity, range of motion, and stability, were prescribed based on participants' functional capacity and fitness level. Participants received continuous support from a program leader and used a journal to track their progress. | | | | | |

| Reference and Country | Title | Author | Study design | Objective | Sample size/age/sex | Assessment instruments | Group | Time/type of intervention | Statistical analysis | Main results | Conclusions | Limitations | Effects on fatigue and dyspnea |
|---|---|---|---|---|---|---|---|---|---|---|---|---|---|
| TORRES, G. et al. | **Exercise interventi** | | Case Study: | To assist in the | A 58-year-old man | The Post-COVID- | - | The exercise | They didn't | The greatest | A combined | - | The greatest |

| | | | | | | | | | | | | | |
|---|---|---|---|---|---|---|---|---|---|---|---|---|---|
| Exercise intervention for post-acute COVID-19 syndrome – do FITT-VP principles apply? A case study. South African Journal of Sports Medicine, [s. l.], v. 35, n. 1, 2023. Disponível em: https://journals.assaf.org.za/index.php/sajsm/article/view/15284. South Africa. [32] | **on for post-acute COVID-19 syndrome – do FITT-VP principles apply? A case study.** | Torres, G. et al., 2023. | Effect of an Exercise Intervention in a Patient with Long-COVID (Applied the Consensus on Exercise Reporting Model). | standardization of exercise for post-COVID, the application of the Consensus on Exercise Reporting Template (CERT) can be very useful. The CERT outlines 16 essential items to ensure clear and complete documentation of exercise interventions. Here are the 16 essential items of the CERT that should be described. | was hospitalized for 14 days in the intensive care unit to treat his severe COVID-19 infection. | 19 Functional Status Scale was administered to assess daily functionality. The Fatigue Severity Scale was used to evaluate fatigue levels, and the MRC Dyspnea Scale was used to assess breathlessness and difficulty breathing. The Hamilton Depression Rating Scale (HAM-D) was utilized to measure depression levels. A submaximal exercise test was | | intervention is described using items from the Consensus on Exercise Reporting Template (CERT): Frequency: 2 supervised sessions and 1 unsupervised walking session per week. Intensity: 65-75% of peak heart rate (HRpeak), Perceived Exertion (PSE): 6-11. Duration: 7-10 minutes. Strength exercises: 1 set of 4-8 repetitions. | mention it, but in the analysis, simple and absolute frequencies were used, as well as comparisons. | improvements were observed in functional status, fatigue, dyspnea, and depression levels. All pulmonary function test variables showed positive changes. Improvements in cardiorespiratory fitness and endurance are clearly demonstrated by changes in peak VO2 and blood lactate threshold. Muscle strength and the blood inflammatory marker | resistance and strength exercise program had positive effects on cardiorespiratory fitness, functional status, fatigue levels, and depression, without adverse effects on parameters that did not show improvement. We recommend using the FITT-VP principles as a useful solution for patients with long-term COVID until more advanced methods are discovered | | improvements occurred in functional status, fatigue, dyspnea, and depression levels. Fatigue Assessment Scale: Pre 27 (fatigue) Post 17 (normal); Medical Research Council Dyspnea Scale: Pre 4* Post 3** % change -25 Absolute -1. |

| | | | | |
|---|---|---|---|---|
| | conducted on a cycle ergometer (Wattbike, Nottingham, England); a handgrip dynamometer (Takei Kiki Kogyo, Japan); a pulse oximeter (Berry BM1000E, Shanghai Berry Electronic Tech Co., Ltd); and venous blood samples were taken for C-Reactive Protein (CRP) and Interleukin-6 (IL-6) analysis. | Walking (Home): 6 to 10 minutes at an RPE of 6 to 11. Type: Resistance exercises: (treadmill, walking, or cycle ergometer). Resistance training: (including balance) using weight machines, dumbbells, stability balls, bands, body weight + flexibility (2-4 exercises per session). Volume: Cannot be defined; needs to be individualized based on the | also improved. | through further research. |

| | | | | |
|---|---|---|---|---|
| | | individual's responses. Progression: Increase walking duration by 1-2 minutes each week; after 3 weeks, increase % of HRpeak by 5% of PSE. Weeks 1-4: PSE 6-9; Weeks 5-8: PSE 9-11. Strength training: 1 set of 4-8 repetitions, increase by 2 repetitions each week. Once reaching 10 repetitions, add 1 set each week up to 3 sets of 10 | | |

| | | | | |
|---|---|---|---|---|
| | | repetitions; also add 1 exercise every 2 weeks. The exercises were based on current exercise prescriptions for patients with long COVID and adapted to the individual based on heart rate responses, PSE, dyspnea, exercise technique, and symptoms of large muscle groups with controlled movement plans (strength equipment), similar to a | | |

| | | | | | | | | | | | | | |
|---|---|---|---|---|---|---|---|---|---|---|---|---|---|
| | | | | | | | | sedentary or beginner resistance training program. | | | | | |

| Reference and Country | Title | Author | Study design | Objective | Sample size/age/sex | Assessment instruments | Group | Time/type of intervention | Statistical analysis | Main results | Conclusions | Limitations | Effects on fatigue and dyspnea |
|---|---|---|---|---|---|---|---|---|---|---|---|---|---|
| DE LA PLAZA SAN FRUTOS, M. et al. Telemedicine in pulmonary rehabilitation – benefits of a Telerehabilitation program in post-COVID-19 patients: a controlled quasi-experimental study. Therapeutic Advances | **Telemedicine in pulmonary rehabilitation – benefits of a telerehabilitation program in post-COVID-19 patients: a controlled quasi-experimental study.** | DE LA PLAZA San Frutos, M. P. et al., 2023. | Quasi-experimental, prospective, longitudinal non-randomized study. April 2020 (presumed until June). | Evaluate the outcomes of a Tele-Rehabilitation Respiratory Program implemented in post-COVID-19 patients in the post-acute phase of mild to critical COVID-19 who exhibited persistent respiratory symptoms and had not received | 148 participants with 100 completing the protocol; the median age of participants was 49 years (IQR = 38–55.75). 69 were women (69%). | Due to mobility limitations caused by confinement, validated questionnaires, scales, and pulse oximetry were used. State anxiety; Trait anxiety; EuroQol-5D quality of life; Mahler dyspnea; Respiratory rate; Oxygen | 2 Groups: n=50 experimental group (GE) and n=50 control group (GC). | Web platform (Zoom by Zoom Video Communications, San Jose, CA, USA): 500 tele-rehabilitation sessions were conducted live; in groups of 20, 3 times a week, totaling 10 online sessions.<br><br>Exercises included: | Expressed in absolute numbers and as a percentage of age; median and interquartile range (IQR); chi-square test (qualitative comparison); Mann-Whitney U test (baseline analysis); Wilcoxon signed-rank test (pre-post analysis); | Significant changes were observed in Mahler's Functional Dyspnea ($p<0.001$); State-Trait Anxiety Inventory ($p<0.001$); oxygen saturation ($p<0.001$); heart rate ($p<0.001$); quality of life questionnaire ($p<0.001$); respiratory rate ($p<0.001$). | Implementation of a pulmonary telerehabilitation program for unvaccinated survivors of post-acute COVID-19 with mild to critical progression and proven respiratory sequelae demonstrated benefits in cardiopulmonary | No disease stratification process was applied; selection of study variables; confined patients could not be moved to assess variables such as exercise capacity, which is widely recommended in telerehabilitation programs. | Significant improvement in Mahler's functional dyspnea ($p < 0.001$); Dyspnea evaluated from 7 to 10 points after the intervention. |

| | | | | | | | | | | | |
|---|---|---|---|---|---|---|---|---|---|---|---|
| in Respiratory Disease, [s. l.], v. 17, p. 175346662311673, 2023. Disponível em: http://journals.sagepub.com/doi/10.1177/175346662311673 54. Madri, Spain. [22] | | | | any vaccination. | | saturation; Heart rate. | | (a) Abdominal-diaphragmatic breathing (b) Costal expansion exercises – upper limb flexion and abduction (c) Active respiratory cycle (d) Self-stretching of neck and thoracic muscles (e) Yoga-based respiratory exercises for pranayama (f) Progressive muscle relaxation (g) Mindfulness. | biserial correlation coefficient (rbis) for effect size, p<0.05. SPSS v.23. | Participants in the control group showed improvement in all variables, but the differences were not statistically significant. | variables and anxiety related to dyspnea. |

| Reference and Country | Title | Author | Study design | Objective | Sample size/age/sex | Assessment instruments | Group | Time/type of intervention | Statistical analysis | Main results | Conclusions | Limitations | Effects on fatigue and dyspnea |
|---|---|---|---|---|---|---|---|---|---|---|---|---|---|
| OSTROWSKA, M. et al. Effects of Multidisciplinary Rehabilitation Program in Patients with Long COVID-19: Post-COVID-19 Rehabilitation (PCR SIRIO 8) Study. Journal of Clinical Medicine, [s. l.], v. 12, n. 2, p. 420, 2023. Disponível em: https://www.mdpi.com/2077- | **Effect of Water-Based vs. Land-Based Exercise Intervention (postCOVIDkids) on Exercise Capacity, Fatigue, and Quality of Life in Children with Post COVID-19 Condition: A Randomized Controlled Trial.** | Ogonowska-Slodownik, A. et al., 2023. | Randomized controlled trial (Warsaw); January 2022 to June 2022. | Analyze the intervention (effectiveness of water-based and land-based training programs) on outcomes such as exercise capacity, fatigue, and secondary outcomes, including health-related quality of life in children with post-COVID-19 condition. | Of the 86 children (aged 10 to 12 years), 74 completed the intervention and attended the post-intervention assessment. | Pediatric Quality of Life Inventory (PedsQL 4.0) and Cumulative Fatigue Symptoms Questionnaire (CFSQ); weight and height for Body Mass Index (BMI); ergometric test; Quality of Life Related to Health (QVRS); treadmill protocol (Modified Balke exercise test). | 3 groups: water exercise (AQUA), land exercise (LAND), or CONTROL (no exercise). | AQUA and LAND sessions were conducted twice a week for eight weeks. The primary outcomes were exercise capacity, measured by the modified Balke treadmill protocol, and fatigue.<br><br>Protocol:<br><br>Frequency: 2 times per week, 45 | Software: Statistica 14. Distribution Assumptions: Since distributions were normal, means were determined using repeated measures analysis of variance (ANOVA).<br>Group Comparisons: Groups were compared using the Kruskal-Wallis test. | Absolute Maximum Oxygen Consumption (VO2max): Increased significantly after AQUA (p = 0,001) and LAND (p = 0,004) interventions. The most significant improvement in VO2max was observed after aquatic physical training. Fatigue: No significant difference | In children aged 10 to 12 years with post-COVID-19 condition, an eight-week supervised physical training program, conducted twice a week either in water or on land, improved exercise capacity. Parents of children in the training groups also noted an improvement in | A heterogeneous group of participants; specific measurements designed and validated in children with post-COVID-19 condition was a limitation in the adequate assessment of fatigue and quality of life; furthermore, the relatively small sample size of the | No significant differences were found between the three groups regarding changes in total fatigue scores or individual fatigue symptoms (rate of complaints) before and after the intervention, according to the CFSQ. |

| | | | | | | |
|---|---|---|---|---|---|---|
| 0383/12/2/420. Poland. [35] | | minutes per session, for 8 weeks (for both intervention groups). Exercises: Aquatic and land exercises were combined to be as similar as possible in terms of intensity, duration, and muscle groups trained. Warm-up: 8 minutes Cool-down: 5 minutes Exercises: Aerobic exercises for upper and lower limbs, including punching and kicking | Pre and Post-Intervention Comparisons: Paired Wilcoxon signed-rank test was used for pre and post-intervention values (equivalent correlation). Between-Group Comparisons: Mann-Whitney test was used for comparisons between groups. Associations: Spearman's correlation was used to assess associations | s were found in total fatigue levels and individual fatigue symptoms between the groups. Quality of Life: A significant improvement in the PedsQL reported by parents was found in the LAND group. These results suggest that both aquatic and land exercises improved exercise capacity, with aquatic training showing a more pronounced effect on | quality of life. | study limits the interpretation of the secondary outcomes. Additionally, the long-term effects of physical exercise were not included in this study, and future research should examine these effects in children with post-COVID-19 condition. |

| | | |
|---|---|---|
| Stationary running Breathing control Stretching for lower limbs Circuit Training: Aerobic Training: 2 circuit stations with resistance exercises for upper and lower limbs, focusing on breathing patterns. Each circuit had 5 stations with different exercises. Each station lasted 1 minute, with 15-second rest intervals between stations. | between variables. Normality Testing: Shapiro-Wilk test. Variance Testing: Levene's test. Post-Hoc Analysis: Tukey’s Honestly Significant Difference (HSD) test for post-hoc comparisons. Effect Sizes: Cohen’s kappa and effect sizes were calculated for ANOVA. | VO2max. Quality of life also significantly improved for the land exercise group, while fatigue levels did not show notable changes. |

| | | | | | | | | | | | | | |
|---|---|---|---|---|---|---|---|---|---|---|---|---|---|
| | | | | | | | | Participants were encouraged to exercise at an intensity of 6 to 8 ("somewhat hard" to "hard") on the Pediatric Rate of Perceived Exertion (PCERT) scale. Cool-down: Stretching for upper limbs and chest, and breathing control. | | | | | |

| Reference and Country | Title | Author | Study Design | Objective | Sample size/age/sex | Assessment instruments | Group | Time/type of intervention | Statistical analysis | Main results | Conclusions | Limitations | Effects on fatigue and dyspnea |
|---|---|---|---|---|---|---|---|---|---|---|---|---|---|
| TAKEKAWA, T. et al. Rehabilitation therapy for a severe | **Rehabilitation therapy for a severe case** | Takekawa, T. et al., 2022. | Case Study. | Determine the Impact of Unsupervised Pulmonary | Male, Japanese, 40 years old. Admitted to the hospital | Computed Tomography; Modified Borg | Single Case. | Individualized rehabilitation, including physical and | - | Improvements in respiratory function tests, Timed Up and Go | We provided rehabilitation therapy to a middle-aged man | - | Our patient was diagnosed with Post-Acute COVID-19 Syndrome |

| | | | | | | | | | | | | | |
|---|---|---|---|---|---|---|---|---|---|---|---|---|---|
| case of coronavirus disease 2019: a case report. Journal of Medical Case Reports, [s. l.], v. 16, n. 1, p. 339, 2022. Disponível em: https://jmedicalcasereports.biomedcentral.com/articles/10.1186/s13256-022-03559-5. Japan. [14] | **of coronavirus disease 2019: a case report.** | | | Rehabilitation. We describe here a nephrectomized patient with severe COVID-19 infection who required extracorporeal membrane oxygenation (ECMO) during admission to the intensive care unit (ICU) but made a full recovery and returned to society after rehabilitation therapy. | with a diagnosis of COVID-19 based on PCR testing. | Scale; Timed Up and Go (TUG) test; 6-Minute Walk Test. | | respiratory rehabilitation therapy. The self-exercise program included exercises 3 times a day, at 10:00, 13:00, and 15:00 hours, for about 30 minutes, 3 sets each time, 6 days per week unsupervised; (1) range of motion joint exercises, (2) muscle strengthening exercises, (3) gait exercises, (4) stair climbing exercises, (5) resistance training, and (6) | | (TUG) test, and 6-minute walk test; reduction in ICU-acquired weakness (ICU-AW) (acquired muscle weakness) . Based on this case, we believe that exercise plays a crucial role in increasing skeletal muscle mass, although nutrition is even more important. | during recovery from a severe COVID-19 infection, focusing on education about nutritional intake, voluntary training, and supervised exercises primarily targeting the trunk and lower limbs. This management improved quality of life (QoL) and facilitated a return to normal activities of daily living (ADLs). | | (PACS) based on the presence of fatigue, muscle weakness, and hair loss, although he did not exhibit other clinical features of the syndrome (e.g., dyspnea, cough, depression, cognitive impairment [brain fog], and palpitations). At discharge, he was completely free of fatigue and muscle weakness. We believe that the muscle weakness in the subacute |

| | |
|---|---|
| self-directed training through supervised and non-equipment-based exercises. Clinical environment (hospitalization) post-discharge, the patient was advised to continue with the voluntary training program, with a subjective intensity of aerobic exercise perceived as “easy,” resistance exercise intensity of 8–12 RM (Repetition Maximum), and | phase of COVID-19 is largely due to acquired weakness in the ICU. |

| | | | | | | | | | | | | | |
|---|---|---|---|---|---|---|---|---|---|---|---|---|---|
| | | | | | | | | without restrictions. | | | | | |

| Reference and Country | Title | Author | Study design | Objective | Sample size/age/sex | Assessment instruments | Group | Time/type of intervention | Statistical analysis | Main results | Conclusions | Limitations | Effects on fatigue and dyspnea |
|---|---|---|---|---|---|---|---|---|---|---|---|---|---|
| JIMENO-ALMAZÁN, A. et al. Effects of a concurrent training, respiratory muscle exercise, and self-management recommendations on recovery from post-COVID-19 conditions: the RECOVE trial. Journal | **Effects of a concurrent training, respiratory muscle exercise, and selfmanagement recommendations on recovery from post-COVID-19 condition: the RECOVE trial.** | Jimeno-Almazán, A. et al., 2022a. | Randomized clinical trial. | Determine the effectiveness of physical exercise, respiratory muscle training, and the World Health Organization (WHO) self-management leaflet on recovery of physical fitness, quality of life, and symptoms. | 80 adults.<br>CT ($n = 21$)<br>CTMR ($n = 25$)<br>RM ($n = 17$)<br>CON ($n = 20$)<br>Average age: 45.3 ± 8.0 years<br>69% female. | Spirometry<br>ECG and Echo<br>Exercise Test (effort test)<br>Body Composition (segmental multifrequency bioelectrical impedance analyzer – Tanita)<br>Heart Rate (HR recorded)<br>Digital Dynamom | CT (Control Treatment);<br>RM (Respiratory Muscle Training);<br>CTRM (Control Treatment with Respiratory Muscle Training);<br>CON (Control) | CT (Control Treatmen)<br>2 supervised resistance training sessions per week<br>Individualized recommendations for aerobic exercises<br>3 days per week:<br>2 days of resistance training (50% 1RM)<br>3 sets of 8 repetitions each | Descriptive Statistics: Calculation of mean, standard deviation (SD), median, interquartile range (IQR), and standard error of the mean (SEM)<br>Normality Test: Shapiro-Wilk test<br>Homogeneity of Variance Test: | VO2max: No significant differences between groups ($P > 0.05$)<br>Significant individual improvements identified in:<br>CT: 7.5% improvement ($P < 0.05$)<br>CTRM: 7.8% improvement ($P < 0.05$) | A multicomponent exercise program has proven to be a safe and effective tool for improving cardiovascular fitness, muscle strength, symptom burden, mood, and quality of life in patients with post-COVID-19 | The limitations include a small sample size; lack of follow-up after the conclusion of the study prevents us from assessing the sustainability of the results; the prognosis of patients with COVID-19 changed | Participants with a score of 10 on the scale showed significant reductions in FSS < 4 (18.8% – 42.5%, $P < 0.001$) and in CSF-Linkert < 18 (28.7% – 57.5%, $P < 0.001$). No significant differences were detected between groups in |

| | | | | | | | | | | | | | |
|---|---|---|---|---|---|---|---|---|---|---|---|---|---|
| of Applied Physiology, [s. l.], v. 134, n. 1, p. 95–104, 2023. Disponível em: https://journals.physiology.org/doi/10.1152/japplphysiol.00489.2022. Spain. [26] | | | | | | eter (Takei 5401-C, Shinagawa-Ku, Tokyo) Sit-to-Stand Test Isometric Knee Extension Test (Chronojump, BoscoSystem, Barcelona) measured in Newtons (N) Submaximal Progressive Load Test (Smith machine) Linear Velocity Transducer (T-Force, Ergotech Consulting, Murcia, Spain) World Health Organization Global | | Exercises: squats, bench press, deadlift, and lat pulldown 1 day of light-intensity continuous training RM (Respiratory Muscle Training) 2 standardized daily sessions 1 set of 30 repetitions [62.5 ± 4.6% of Maximum Inspiratory Pressure (MIP)] Preceded by a warm-up set CTRM (Combination of Control Treatment and Respirator | Levene's test Inferential Statistics: Two-way factorial analysis of variance (ANOVA) for group and time (pre vs. post) Post-hoc Bonferroni adjustment One-way ANOVA McNemar test for categorical data Effect Size: Cohen's d for factorial analyses Cohen's g for McNemar analyses Significance Level: $p < 0.05$ Software: | Lower Body Strength: Significant improvements in multicomponent training (14.5–32.6% improvement, $P < 0.05$) No changes in RM and CON groups Fatigue Scores: Significant decrease in Fatigue Severity Scale (FSS) < 4 (18.8% – 42.5% improvement, $P < 0.001$) Significant decrease in Chalder Fatigue Scale (CFS) Linkert < | conditions. It requires minimal costs and resources. Self-care and information provided are highly inefficient and are not an effective treatment alternative when used in isolation. | over the course of the study due to new treatments and vaccines, and it is unclear whether vaccines have an inducing or relieving effect on symptoms once the syndrome develops; other factors such as nutritional and hydration status, sleep and rest time, medications, and other behavioral factors were not controlled. | mMRC (dyspnea). |

| | | | | | | | | | | | | | |
|---|---|---|---|---|---|---|---|---|---|---|---|---|---|
| | | | | | | Physical Activity Questionnaire (GPAQ)<br>Forced Spirometry (MetaLyzer 3B-R3, Cortex Biophysik GmbH, Leipzig, Germany)<br>Forced Vital Capacity (FVC)<br>Maximum Voluntary Ventilation (MVV)<br>Perceived Exertion (PSE) (according to the modified Borg scale)<br>PROM<br>Short Form Survey (SF-12)<br>Generalized Anxiety Disorder-7 (GAD-7) | | y Muscle Training)<br>Combination of CT and RM interventions<br>CON (Control)<br>Received a leaflet with recommendations from the World Health Organization (WHO)<br>No additional supervised exercise sessions. | SPSS version 25.0. | 18 (28.7% – 57.5% improvement, P < 0.001) | | | |

| | | | | | | | | | | | | | |
|---|---|---|---|---|---|---|---|---|---|---|---|---|---|
| | | | | | | Patient Health Questionnaire-9 (PHQ-9) Dyspnea Estimation using the Modified Medical Research Council Dyspnea Scale (mMRC) Chalder Fatigue Scale (CFS) | | | | | | | |
| Reference and Country | Title | Author | Study design | Objective | Sample size/age/sex | Assessment instruments | Group | Time/type of intervention | Statistical analysis | Main results | Conclusions | Limitations | Effects on fatigue and dyspnea |
| LONGOBARDI, I. et al. Benefits of Home-Based Exercise Training Following Critical SARS-CoV-2 Infection: A Case | **Benefits of Home-Based Exercise Training Following Critical SARS-CoV-2 Infection: A Case Report.** | Longobardi et al., 2022. | Case report. | To recover overall physical condition. | 67-year-old woman. | Cardiopulmonary parameters, skeletal muscle strength and functionality, severity of fatigue, and self-reported persistent symptoms | - | 10 weeks; HBET (home-based exercise training) semi-supervised. Two sets of 10 minutes/day walking at a "very | - | Notable improvements in peak VO2; oxygen consumption efficiency slope; lower VE/VCO2 ratio; reduced exertional | A semi-supervised HBET program can be safe and potentially effective in improving cardiorespiratory and physical functionali | Inherent limitations of a case study, the data reported here are encouraging and may help pave the way for randomized clinical | Isothermic exertional dyspnea was reduced, and Fatigue (FSS) decreased from 4 to 2.7. |

| | | | | | | | | | | |
|---|---|---|---|---|---|---|---|---|---|---|
| Report. Frontiers in Sports and Active Living, [s. l.], v. 3, p. 791703, 2022. Disponível em: https://www.frontiersin.org/articles/10.3389/fspor.2021.791703/full. Brazil. [27] | | | | | | ; (Cardiopulmonary Exercise Test, CPET); handgrip test, 30-second sit-to-stand test (30-STS), and Timed-Up-and-Go (TUG) test; post-COVID functional status; fatigue severity was assessed using a specific scale. | light” to “fairly light” intensity (Borg scale 9–11). Progressed to a single 45-minute walking session at a “somewhat hard” to “hard” intensity (Borg scale 14–16). Six strengthening exercises, with strengthening training sessions comprising 3 to 4 sets per exercise of 10 to 15 repetitions, with a self-suggested recovery interval | dyspnea; Fatigue Severity Scale (FSS) score reduced from 4 to 2.7. | ty in COVID-19 survivors. | trials testing the safety, efficacy, and feasibility of exercise interventions as an adjunct. |

| | | | | | | | | | | | | | |
|---|---|---|---|---|---|---|---|---|---|---|---|---|---|
| | | | | | | | | between sets, starting at “very light” to “fairly light” intensity (Borg scale 9–11) and progressing towards “somewhat hard” to “hard” (Borg scale 14–16). Active stretching exercises for major muscle groups were prescribed as relaxation. | | | | | |

| Reference and Country | Title | Author | Study design | Objective | Sample size/age/sex | Assessment instruments | Group | Time/type of intervention | Statistical analysis | Main results | Conclusions | Limitations | Effects on fatigue and dyspnea |
|---|---|---|---|---|---|---|---|---|---|---|---|---|---|
| MÁRQUEZ-SILVA, | **Application of a** | | Quasi-experimen | Generate an | 5 patients, ranging | | 1 group. | 10 Sessions: | - | In the Fatigue | Physical activity in | Inherent limitations | On the Fatigue |

| | | | | | | | | | | | | | |
|---|---|---|---|---|---|---|---|---|---|---|---|---|---|
| S. et al. Application of a therapeutic exercise program to improve physical condition in patients with post-COVID-19 syndrome. Revista de Técnicas de la Enfermería y Salud, [s. l.], p. 1–8, 2022. Disponível em: https://www.ecorfan.org/republicofperu/research_journals/Revista_de_Tecnicas_de_la_Enfermeria/vol6num16/Journal_of_Nursing_Techniques_V6_N16_1.pdf. | **therapeutic exercise program to improve physical condition in patients with post-COVID-19 syndrome.** | Márquez-Silva et al., 2022. | tal quantitative. | exercise program to improve the physical condition of patients with post-COVID-19 syndrome. | from 18 years old to the oldest patient at 56 years old. | Borg/6-minute walk test Oximetry Barthel Index Fantastic Questionnaire Fatigue Assessment Scale (FAS) Modified Medical Research Council Scale (mMRC) Single breath count. | | Frequency: Four times per week Duration: 60 to 90 minutes per session Session Breakdown: Warm-up Main Phase: Strength and muscle resistance exercises Aerobic training Balance exercises Respiratory exercises (Self-developed). | | Assessment Scale (FAS): Initial Mental Assessment Score: Minimum score of 13. Results: Significant improvement in physical condition. | the context of a pandemic can help: Maintain or improve exercise tolerance, Maximal oxygen consumption, Functional capacity, Cardiovascular health, Body composition, Muscle strength. | of a case study: The data reported here are encouraging and may help pave the way for randomized clinical trials testing the safety, efficacy, and feasibility of exercise interventions as adjuncts. | Assessment Scale (FAS): Initial mental evaluation: Minimum score was 13 and maximum score was 24. Reevaluation: Minimum score was 11 and maximum score was 18. Mental FAS showed a decrease of 3.6, and physical FAS showed a decrease of 4.4. |

| | | | | | | | | | | | | | |
|---|---|---|---|---|---|---|---|---|---|---|---|---|---|
| Mexico. [33] | | | | | | | | | | | | | |
| Reference and Country | Title | Author | Study design | Objective | Sample size/age/sex | Assessment instruments | Group | Time/type of intervention | Statistical analysis | Main results | Conclusions | Limitations | Effects on fatigue and dyspnea |
| MAYER, K. P. et al. Physical Therapy Management of an Individual With Post-COVID Syndrome: A Case Report. Physical Therapy, [s. l.], v. 101, n. 6, p. pzab098, 2021. Disponível em: https://academic.oup.com/ptj/article/doi/10.1093/ptj/ | **Physical Therapy Management of an Individual With Post-COVID Syndrome: A Case Report.** | Mayer, K. P. et al., 2021. | Case report. | Provide the Clinical Presentation and Physiotherapeutic Management of a Patient with Post-COVID Syndrome. | Woman 37 years old. | Battery of tests, including muscle strength, lower limb muscle power, exercise capacity tests, cognitive tests, and emotional health outcomes; raw scores and reference values.<br><br>Timed-Up and Go (TUG) test Impact of Events Scale- | - | Biweekly sessions, for 8 weeks (16 sessions) including aerobic training, strength exercises, diaphragmatic breathing techniques, and mindfulness training.<br><br>Aerobics: Recumbent upper-limb ergometer, stationary bike, running, and dancing. | - | Muscle strength, physical function, and exercise capacity of the patient improved. After physiotherapy, the patient still experienced headaches, dyspnea, fatigue, and cognitive dysfunction. | There is limited evidence to guide rehabilitation assessments and interventions. Physical therapists should consider cognitive function and emotional health in their care plans for patients with post-COVID syndromes. The treatment program | - | Excessive exertion led to discomfort and an increase in fatigue frequency. |

| | | | |
|---|---|---|---|
| pzab098/6177704. U.S.A. [15] | Revised (IES-R) mMRC – dyspnea PSEm Post-Traumatic Stress Disorder (PTSD) Visual Analog Scale EQ-5D-5L. | Strength training was prescribed based on the PSEm, with an initial rating of 5 to 6/10, performing 10 to 15 repetitions. 10 to 20 minutes of resistance training with exercises focusing on major muscle groups, including multi-joint or compound exercises (e.g., leg press). Functional movements according to progression. | was associated with improved physical performance but limited improvements in emotional health and cognitive function. |

| Reference and Country | Title | Author | Study design | Objective | Sample size/age/sex | Assessment instruments | Group | Time/type of intervention | Statistical analysis | Main results | Conclusions | Limitations | Effects on fatigue and dyspnea |
|---|---|---|---|---|---|---|---|---|---|---|---|---|---|
| SARİ, F. et al. Effects of Inspiratory Muscle Training in Patients with post-COVID-19. Harran Üniversitesi Tıp Fakültesi Dergisi, [s. l.], v. 19, n. 3, p. 581–588, 2022. Disponível em: http://dergipark.org.tr/en/doi/10.35440/hutfd.1136549. Turkey. [28] | **Effects of Inspiratory Muscle Training in Patients with post-COVID-19.** | Sari, F. et al., 2022. | Prospective, double-blind, randomized, and controlled clinical study.<br>March to June 2021. | Investigate the efficacy of inspiratory muscle training on exercise capacity, reduced muscle strength, dyspnea, anxiety-depression, quality of life, physical activity, and fatigue in patients with Coronavirus Disease 2019 (COVID-19) and respiratory illnesses. | 24 participants aged 18 to 65 years (10 men and 3 women) and 11 in the control group (6 men and 5 women). | Functional Capacity: 6-Minute Walk Test (6-MWT); Sit-to-Stand Test (30 seconds); Dyspnea: Modified Medical Research Council (MMRC) Dyspnea Scale, comprising 5 items on dyspnea, scored between 0-4;<br><br>Handgrip Strength: Digital hand dynamometer (J-Tech™, Midvale, USA); | 2 Groups: Treatment Group (GT) Control Group (GC). | The respiratory exercises included:<br>Diaphragmatic breathing<br>Thoracic expansion<br>Exercises to increase thoracic compliance with respiratory control using an exercise band.<br>The respiratory exercises were performed:<br>For 5 to 10 repetitions<br>1 set of 5 to 10 minutes<br>3 sets/day<br>The resistance | Software used:<br>G-Power (version 3.1.9.2)<br>SPSS 15.0<br>Statistical methods:<br>Variables with non-normal distribution were expressed as median (25-75 interquartile range [IQR]) and compared using the Mann-Whitney U test.<br>The Wilcoxon Signed Rank test was used for intragroup comparisons. | Improvements in the treatment group (GT) were statistically more significant than in the control group (GC).<br>The 6-Minute Walk Test (TC6) distance increased significantly in the GT ($p < 0.001$).<br>When analyzing the effect of the treatment between groups, there was a significant improvement in the | Inspiratory Muscle Training (IMT) as a useful, practical, and safe exercise method increased exercise capacity and decreased muscle strength while reducing the perception of dyspnea, anxiety, and depression in post-COVID-19 patients over 6 weeks. However, IMT did not show an effect | Respiratory muscle strength/resistance and pulmonary function tests were not evaluated, which prevented us from assessing differences in these measures between groups after treatment. The inspiratory muscle strength program was supervised for only 6 weeks, which is a short period to observe | Dyspnea and fatigue decreased in both groups ($p < 0.05$).<br>After treatment, 2 (15.38%) patients in the treatment group (GT) experienced dyspnea, while 6 (54.54%) patients in the control group (GC) experienced dyspnea.<br>The difference between the two groups was significant after treatment ($p < 0.05$). |

| | | | | | | | | | | | | |
|---|---|---|---|---|---|---|---|---|---|---|---|---|
| | | | | | | Anxiety and Depression: Hospital Anxiety and Depression Scale (HADS); Physical Activity Level: International Physical Activity Questionnaire (IPAQ); Fatigue: Fatigue Severity Scale (FSS), consisting of 9 items. A higher total score indicates worse perception of fatigue. | | training included: Quadriceps strengthening exercises such as squats Clinical bridge exercises based on Pilates. The resistance exercises were performed: For 6 weeks Every day 10 repetitions 3 sets/day Approximately 10 minutes. | Significance level: p<0.05. | GT compared to the GC (p < 0.05). Significant improvements were observed in the 30-second Sit-to-Stand Test. No significant changes were demonstrated in the Modified Medical Research Council (MMRC) dyspnea scale between GT and GC (p = 0.87). The MMRC dyspnea scale decreased significantly within the GT after | on quality of life, physical activity, or fatigue in our study. | treatment effects. We did not have a third control group that did not receive any rehabilitation intervention. Cardiopulmonary exercise tests were not applied in our study. |

| | | | | | | | | | | | | | |
|---|---|---|---|---|---|---|---|---|---|---|---|---|---|
| | | | | | | | | | | treatment ($p < 0.05$). | | | |

| Reference and Country | Title | Author | Study design | Objective | Sample size/age/sex | Assessment instruments | Group | Time/type of intervention | Statistical analysis | Main results | Conclusions | Limitations | Effects on fatigue and dyspnea |
|---|---|---|---|---|---|---|---|---|---|---|---|---|---|
| STAVROU, V. T. et al. Eight Weeks Unsupervised Pulmonary Rehabilitation in Previously Hospitalized of SARS-CoV-2 Infection. Journal of Personalized Medicine, [s. l.], v. 11, n. 8, p. 806, 2021. Disponível em: https://www.mdpi.com/2075- | **Eight Weeks Unsupervised Pulmonary Rehabilitation in Previously Hospitalized of SARS-CoV-2 Infection.** | Stavrou V. T. et al., 2021. | Exploratory Design | Determine the impact of Unsupervised Pulmonary Rehabilitation (uns-PR) in patients recovering from COVID-19, and determine their anthropometric and biological characteristics.. | (n=20, age: 64.1 ± 9.9 years, 75% male). | 6-minute walk test (6MWT); blood sampling for oxidative stress measurement before and after unsupervised pulmonary rehabilitation (uns-PR); resting systolic blood pressure; heart rate; oxygen saturation; dyspnea at the end of the 6MWT; | 1 group (intervention). | 8 weeks;<br>3 training sessions per week;<br>Each session about 100 minutes;<br>1 warm-up series 5';<br>1 recovery series 5';<br>Flexibility and mobility exercises;<br>Aerobic exercise series with walking 50';<br>Yoga series for breathing and/or proprioception 20'; | Data are presented as mean ± SD and frequency (%) where appropriate;<br>Kolmogorov-Smirnov test;<br>paired sample t-test; ($p < 0.05$). | We observed differences before and after unsupervised pulmonary rehabilitation (uns-PR) during the 6-Minute Walk Test (6MWT) in the following parameters:<br><br>Hemodynamic Parameters:<br>Resting systolic blood pressure: | Unsupervised pulmonary rehabilitation can be an effective and beneficial practice for promoting exercise and recovery from symptoms following post-COVID-19, as well as a new approach for addressing persistent fatigue induced | Sample size; a negative control group was not considered; we did not account for multiple comparison adjustments; although statistically significant, specific results may not be translated meaningfully into clinical practice. | Unsupervised pulmonary rehabilitation can be an effective practice and a new approach for treating persistent fatigue (improvement, though not significant, $p>0.05$) induced by SARS-CoV-2 infection. Post-exercise dyspnea shows significant improvem |

| | | | | | | | |
|---|---|---|---|---|---|---|---|
| 4426/11/8/806. Greece. [29] | | | distance walked; estimated O2; 30-second sit-to-stand test; self-assessment of fatigue and dyspnea in the lower limbs were captured through the Borg CR10 scale; body composition parameters; and sleep quality (Pittsburgh Sleep Quality Index [PSQI]). | Multi-joint strength exercises series 20’; Nutritional recommendations. | 138.7 ± 16.3 vs. 128.8 ± 8.6 mmHg, p = 0.005 End of test systolic blood pressure: 159.8 ± 13.5 vs. 152.0 ± 12.2 mmHg, p = 0.025 Heart rate at 5th minute: 111.6 ± 16.9 vs. 105.4 ± 15.9 bpm, p = 0.049 Heart rate at 6th minute: 112.5 ± 18.3 vs. 106.9 ± 17.9 bpm, p = 0.039 Oxygen Saturation: 4th minute: 94.6 ± 2.9 vs. 95.8 ± | by SARS-CoV-2 infection. | ent (p<0.05). |

| | | | | | | |
|---|---|---|---|---|---|---|
| | | | | | 3.2%, p = 0.013<br>1st minute of recovery:<br>97.8 ± 0.9 vs. 97.3 ± 0.9%<br>Dyspnea:<br>End of 6MWT:<br>1.3 ± 1.5 vs. 0.6 ± 0.9 score, p = 0.005<br>Distance Walked:<br>433.8 ± 102.2 vs. 519.2 ± 95.4 meters, p < 0.001<br>Estimated VO2:<br>14.9 ± 2.4 vs. 16.9 ± 2.2 mL/min/kg, p < 0.001<br>30-Second Sit-to-Stand Test:<br>11.4 ± 3.2 vs. 14.1 ± 2.7 repetitions, p < 0.001 | |

| |
|---|
| Plasma Antioxidant Capacity: 2528.3 ± 303.2 vs. 2864.7 ± 574.8 U.cor., p = 0.027<br>Body Composition Parameters:<br>Body fat: 32.2 ± 9.4 vs. 29.5 ± 8.2%, p = 0.003<br>Visceral fat: 14.0 ± 4.4 vs. 13.3 ± 4.2 score, p = 0.021<br>Neck circumference: 39.9 ± 3.4 vs. 37.8 ± 4.2 cm, p = 0.006<br>Muscle mass: 30.1 ± 4.6 vs. 34.6 ± 7.4 kg, p = 0.030 |

| | | | | | | | | | | | | | |
|---|---|---|---|---|---|---|---|---|---|---|---|---|---|
| | | | | | | | | | | Sleep Quality: 6.7 ± 3.9 vs. 5.6 ± 3.3, $p = 0.036$. | | | |
| Reference and Country | Title | Author | Study design | Objective | Sample size/age/sex | Assessment instruments | Group | Time/type of intervention | Statistical analysis | Main results | Conclusions | Limitations | Effects on fatigue and dyspnea |
| WAHYUNI, L. K. et al. The Influence and Feasibility of Therapeutic Exercise Videos at Home on the Functional Status of Post-COVID-19 Hospitalization. Kesmas: Jurnal Kesehatan Masyarakat Nasional, [s. l.], v. | **The Influence and Feasibility of Therapeutic Exercise Videos at Home on the Functional Status of Post-COVID-19 Hospitalization.** | Wahyuni, L. K. et al., 2023. | Quasi-experimental study Multicentric with randomized sampling without blinding. August 2021 to March 2022. | Analyze the effect of educational videos on therapeutic exercises on the functional status of subjects after hospitalization for COVID-19. | 27 pacients. | 6-minute walk test (6MWT), oxygen saturation, Borg scale; 30-second sit-to-stand test (30s STS); Fatigue Severity Scale (FSS); Barthel Index (functional independence); method feasibility assessed using the Technolog | 2 groups: control and intervention. | 1 week; Control: Conventional education on home therapeutic exercises; Intervention: Educational videos on home therapeutic exercises 2-3 times a day with 5–10 repetitions for each movement. Exercise: Balance and | Software: SPSS 22.0; Analysis: Univariate analysis (participant characteristics and all variables); 6-Minute Walk Test (TC6) in meters; 30-Second Sit-to-Stand Test (STS) in counts; Fatigue Severity Scale (FSS) and Barthel | Functional improvements after one week of intervention in both groups. Control group: No statistically significant improvement. Intervention group: Significant improvement in all outcomes. | Significant functional improvement can be observed after one week of therapeutic exercise following video-based instructions at home compared to conventional methods. Video-based tele-rehabilitation is feasible and can | Limitations include a small number of subjects; consecutive sampling (which may not represent the general COVID-19 population); and lack of long-term measurement. | There was no significant difference for FSS ($p = 0.611$). |

| | | | | | | | | | | | | | |
|---|---|---|---|---|---|---|---|---|---|---|---|---|---|
| 18, n. sp1, p. 43, 2023. Disponível em: https://scholarhub.ui.ac.id/kesmas/vol18/iss5/8. Indonesia. [34] | | | | | | y Acceptance Model (TAM) questionnaire. | | strengthening (seated, phase 1): Balance and strengthening with support/active weight including sit-to-stand and alignment or walking assistance. Phase 2: Further progression in balance and strengthening. | Index by scores; Paired t-test; Wilcoxon test; Data presented as mean ± SD; Significance level: $p < 0.05$. | | enhance the functional status of hospitalized post-COVID-19 patients. | | |
